# Occupational groups and its physical and mental health correlates: results from the Singapore Mental Health Study 2016

**DOI:** 10.1007/s00420-021-01741-8

**Published:** 2021-07-24

**Authors:** Rajeswari Sambasivam, Anitha Jeyagurunathan, Edimansyah Abdin, Saleha Shafie, Sherilyn Chang, Janhavi Ajit Vaingankar, Siow Ann Chong, Mythily Subramaniam

**Affiliations:** Research Division, Institute of Mental Health, Singapore, Buangkok Green Medical Park, 10 Buangkok View, Singapore, 539747 Singapore

**Keywords:** Occupational groups, Physical conditions, Mental wellbeing, Psychosocial

## Abstract

**Purpose:**

The physical and mental wellbeing of an individual is impacted by the type occupation one does. This study aims to establish the prevalence of mental and physical disorders, the association of occupational groups and health-related quality of life, and the extent of work-loss and work-cut back in past 30 days among the employed in the Singapore resident population.

**Methods:**

Data from a population-based, epidemiological survey of a representative sample of Singapore citizens and permanent residents aged 18 years and above were used. Lifetime diagnosis of select mental disorders was established using the World Health Organization’s Composite International Diagnostic Interview version 3.0 (WHO-CIDI 3.0). Data on nicotine dependence, work productivity, quality of life and socio-demographics were obtained via self-report. Ten major occupational groups based on the Singapore Standard Occupational Classification were included in the analysis.

**Results:**

The sample comprised 4021 employed individuals who were predominantly males (54.7%) and aged 35–49 years (35.4%). ‘Service and sales workers’ (22.6%), ‘Professionals’ (17.3%) and ‘Legislators, senior officials and managers’ (16.4%) were the three largest occupational groups. Socio-demographic characteristics differed significantly (*p* < 0.001) across all occupational groups. Lifetime prevalence of mood disorders among the employed was 8.4% and the most prevalent physical disorder was chronic pain (18.9%). No significant differences were observed in work productivity loss across the occupational groups.

**Conclusions:**

The disparities in the socio-demographic characteristics and prevalence of mental and physical disorders across occupational categories provide policymakers with vital information to pilot effective interventions that can improve the psychosocial and physical conditions at work.

## Introduction

Majority of people’s time is spent working and thus the workplace “is one of the key environments that affect their mental wellbeing and health” (World Health Organization 2000). The effect of having an occupation may be a double-edged sword; creating a positive impact on one’s wellbeing by providing a sense of personal identity and financial security whilst on the other hand it may cultivate pressure due to the increasing demands of modern-day work life, which, in turn affects the physical and mental wellbeing of an individual (International Labour Organization 2016).

Studies conducted across European and North American countries have shown that different types of occupations are associated with high rates of mental disorders; people involved in occupations such as sales, services, clerical, teaching, welfare workers, cooking seem to be particularly at risk (Stansfeld et al. 2011). Studies of psychosocial work characteristics and mental health revealed that job strain leads to increased risk of depression and other common mental disorders (Stansfeld and Candy 2006; Netterstrom et al. 2008). It has also been widely established that physical conditions such as asthma and other respiratory symptoms (Dodd and Mazurek 2016; Schyllert et al. 2018) differ in prevalence among occupational groups. Studies have shown that workplace productivity loss is associated with physical and mental health conditions (Boles et al. 2004; Mitchell and Bates 2011). Holden (2011) recognized that psychological distress when present as a co-morbid condition determines an increased risk of productivity loss for a variety of health conditions.

Singapore is a country in Southeast Asia with a total population of about 6 million. Chinese (74.4%) form the majority of the population, followed by Malays (13.4%), Indians (9.0%) and those from other ethnic groups (3.2%) (SingStat 2019). In 2019, 80.8% of Singapore resident population aged 25–64 years was employed. Of these, 58.3% were in professionals, managerial, executive and technical (PMET) occupational group, 22.2% were in clerical, sales and service workers group and 19.5% made up the production and transport operators, cleaner and labourers occupational group (Ministry of Manpower 2019). Using data from the Singapore Mental Health Study, a population-wide epidemiological study conducted in Singapore in 2010, Vaingankar et al. (2015) reported that the lifetime prevalence of having ‘any physical disorder’ and ‘any mental disorder’ among those employed, were 37.9% and 13.0% respectively, with hypertension being the most prevalent physical disorder (15.6%) and major depressive disorder being the most prevalent mental disorder (5.9%).

Following the completion of the second Singapore Mental Health Study conducted in 2016 (henceforth referred to as SMHS 2016) (Subramaniam et al. 2019), this present study set out to establish (1) the prevalence of mental and physical disorders among the employed in Singapore, (2) the association of occupational groups and health-related quality of life, and (3) the extent of work-loss and work-cut back in past 30 days in the Singapore resident population.

## Methods

### Survey population and procedure

Data for this study were gathered from the SMHS 2016; a population-based, epidemiological study conducted between 2016 and 2018. The study was approved by the National Healthcare Group Domain Specific Review Board (NHG DSRB). Survey respondents were randomly selected (*N* = 15,900) from a national database of Singapore citizens and permanent residents aged 18 years and above (*N* = 311,687). Residents who were institutionalised or hospitalised for the duration of the study, unable to do the interview due to severe physical or mental illness, those living outside of Singapore, and those who were unable to communicate either in English, Mandarin or Malay were excluded from the study. A disproportionate stratified sampling design was used to over-sample residents aged 65 and above, and those of Malay and Indian ethnicity to ensure that sufficient samples sizes were achieved to improve the reliability of estimates for these groups. An invitation letter was sent to each resident which was followed by an interviewer visiting their homes to obtain their written informed consent to participate in the study. Trained interviewers from a survey research firm conducted the face-to-face interviews with residents who agreed to participate in the study. Further methodological details can be found in an earlier paper (Subramaniam et al. 2019).

The overall survey response rate was 69.5% and a total of 6126 respondents participated in the study. Respondents were queried about their employment situation during the time of the survey by asking them to indicate if they were “working now for pay, self-employed, looking for work, disabled, temporarily laid off, retired, a home-maker, a full-time or part-time student or, have never worked”. Respondents then had to also specify their occupation and the industry they worked in. The employment level in Singapore ranged from 67.7% to 68.3% between 2015 and 2019 (Ministry of Manpower Singapore 2020). Data from 4021 employed respondents were categorized into the following employment groups according to ten major occupational groups based on the Singapore Standard Occupational Classification (SSOC 2020): 1. Business owners, 2. Legislators, senior officials and managers, 3. Professionals, 4. Associate professionals and technicians, 5. Clerical support workers, 6. Service and sales workers, 7. Craftsmen and related trades workers, 8. Plant and machine operators and assemblers, 9. Cleaners, labourers and related workers and 10. Agricultural and fishery workers. The classification of the occupational groups followed that of earlier research in this area (Vaingankar et al. 2015). The occupational group ‘Agricultural and fishery workers’ (*n* = 3) was excluded from the analysis due to low sample size.

### Mental disorders

The fully structured computer-assisted personal interview version of the Composite International Diagnostic Interview version 3.0 (WHO-CIDI 3.0) (Kessler and Ustün 2004) is designed to generate diagnostic information according to definitions and criteria of the Diagnostic and Statistical Manual of Mental Disorders, 4th Edition (DSM-IV) (APA 1994) and the International Classification of Disease, 10th Revision (ICD-10), Classification of Mental and Behavioral Disorders (WHO 1992). Select diagnostic modules of the WHO-CIDI 3.0, major depressive disorder (MDD), bipolar disorder (BD), generalized anxiety disorder (GAD), obsessive compulsive disorder (OCD), and alcohol use disorder (AUD) which includes alcohol abuse and alcohol dependence, were administered to the respondents to reduce respondent burden. For this study MDD and BD were classified as mood disorders and GAD and OCD as anxiety disorders.

### Chronic physical conditions

Interviewers read out a list of 18 chronic physical conditions and asked participants to report if the latter were ‘ever told by a Doctor’ that they had any of these conditions. These chronic conditions included hypertension, hyperlipidaemia, diabetes, asthma, chronic pain (arthritis or rheumatism, back problems such disk or spine problems, and migraine headaches), cardiovascular disorders (stroke or major paralysis, and heart disease including heart attack, coronary heart disease, angina and congestive heart failure), ulcers and chronic inflamed bowel problems such as enteritis or colitis, thyroid diseases, cancer, kidney failure, neurological disorders (epilepsy, convulsions and Parkinson’s disease) and chronic lung diseases (chronic bronchitis or emphysema). In this article, we have excluded cardiovascular disorders, ulcers and chronic inflamed bowel problems, thyroid diseases, cancer, kidney failure, neurological disorders and chronic lung diseases due to their low prevalence i.e., less than 5.0% (Schäfer 2012) in the population. Hypertension, hyperlipidaemia, diabetes, asthma and chronic pain (arthritis or rheumatism, back problems such disk or spine problems, and migraine headaches) have been included for the purpose of this study.

### Nicotine dependence

The 6-item modified Fagerstrom Test for Nicotine Dependence was used to assess the intensity of physical dependence on nicotine. Scores of 4 and less are categorised as low dependence and scores of 8–10, as high dependence. As defined by a previous study (Shahwan et al. 2019), we classified those with scores 5 and above as dependence.

### Work productivity

The effect of mental and physical disorders on work productivity was assessed using the 30-day functioning module of the WHO-CIDI 3.0. The number of days when respondents were totally unable to work or carry out their regular activities, or had to cut back on the quality and quantity of their work due to problems with their physical health, mental health, or use of alcohol or drugs in the month preceding the survey were reported as work-loss and work-cut-back days respectively.

### Health-related quality of life

The Short Form 12-item (SF-12) Health Survey Questionnaire is an abbreviated version of the SF-36, and is commonly included in population-based studies to assess perceived health status (Ware et al. 1996), and in clinical and community settings (SF-12, 2002). Thus, the SF-12 measures eight concepts commonly represented in widely used surveys: physical functioning (PF), role limitations due to physical health problems (RP), bodily pain (BP), general health (GH), vitality (energy/fatigue) (VT), social functioning (SF), role limitations due to emotional problems (RE), and mental health (psychological distress and psychological wellbeing) (MH). The developers have suggested obtaining summary scores from the instrument, the intention being to reduce the original eight-scale profile to two summary measures without substantial loss of information. The summary measures were constructed independently to reproduce corresponding SF-12 physical and mental health summary measures. Scores ranged from 1 (the worst possible health) to 100 (the best possible health). The physical component summary scores (PCS) and mental component score (MCS) have evidence of reliability, validity and responsiveness in population studies (Ware et al. 1996).

### Socio-demographic information

Respondents’ socio-demographic information on age, gender, ethnicity, marital status, highest education level, average household income and employment status were obtained via a structured questionnaire.

### Statistical analysis

All estimates were weighted to adjust for over sampling, non-response and post-stratified for age and ethnicity distributions between the survey sample and the Singapore resident population in 2014 to ensure that the survey findings were representative of the Singapore adult population. Descriptive analyses were performed to describe the socio-demographic profile of the study population and lifetime prevalence of physical and mental disorders across occupational groups. Associations between occupational groups and other categorical variables were examined using χ^2^ test and multivariable logistic regression analyses. The multicollinearity between independent variables was analyzed using a variance inflation factor (VIF) command, with a VIF value of more than 10 considered as indicative of multicollinearity. We found that the VIF values ranged from 1.07 to 3.03. Statistical significance was set at *p* < 0.05 and all statistical analyses were performed using the Statistical Analysis Software (SAS) system version 9.3 (Cary, NC, USA). Missing values in this dataset were handled using the listwise method.

## Results

### Occupation and socio-demographic characteristics

Four thousand and twenty-one employed individuals were included in this study sample. Majority of the participants were males (54.7%), aged 35–49 years (35.4%), of Chinese ethnicity (75.9%), married (62.5%), attained university education level (35.1%), with a household income of $6000–9999 (24.1%) (Table 1). The top three occupational groups were 1. Service and sales workers (22.6%), 2. Professionals (17.3%) and 3. Legislators, senior officials and managers (16.4%). Age group, gender, ethnicity, marital status, educational level and household income showed significant (*p* < 0.001) differences across all occupational groups (Table 2). The most common occupational group among the older aged participants (50 years and above) was ‘Cleaners, Labourers and Related Workers’ whilst ‘Legislators, senior officials and managers’ and ‘Professionals’ were the most common occupations among those aged 35–49 and 18–34 years, respectively.

**Table 1:** Socio-demographic characteristics of the respondents

	*N* (4021)	%
Age group		
18–34	1219	30.9
35–49	1246	35.4
50–64	1178	28.0
65 +	378	5.8
Gender		
Male	2303	54.7
Female	1718	45.3
Ethnicity		
Chinese	1212	75.9
Malay	1249	12.2
Indian	1228	8.7
Others	332	3.3
Marital status		
Never married	1041	30.2
Married	2630	62.5
Divorced/separated	250	5.6
Widowed	100	1.8
Education		
Primary and below	508	11.9
Secondary	966	19.9
Pre-U/Junior College	166	4.8
Vocational Institute/ITE	405	7.3
Diploma	805	21.0
University	1171	35.1
Household Income (SGD)		
Below 2000	514	10.4
2000–3999	930	19.7
4000–5999	845	22.7
6000–9999	782	24.1
10,000 and above	698	23.1

*SGD *Singapore Dollar

**Table 2:** Occupation and socio-demographic distribution of the sample (*n* = 4021)

	All occupation (4021)	Legislators, senior officials and managers (658)	Professionals (696)	Associate professionals and technicians (649)	Clerical support workers (365)	Service and sales workers (909)	Craftsmen and related trades workers (89)	Plant and machine operators and assemblers (392)	Cleaners, labourers and related workers (214)	Business owners (49)
Overall sample (%)	–	16.4	17.3	16.1	9.1	22.6	2.2	9.7	5.3	1.2
Age group*										
18–34	30.9	25.7	44.1	36.0	33.5	33.3	20.5	13.9	7.7	20.7
35–49	35.4	49.7	36.5	33.4	38.8	26.4	33.4	29.6	16.9	37.9
50–64	28.0	22.9	16.8	24.6	24.1	32.3	40.9	47.7	51.9	29.6
65 +	5.8	1.7	2.6	6.1	3.6	8.0	5.2	8.6	23.5	11.8
Gender*										
Male	54.7	56.8	52.4	59.7	21.7	54.1	76.6	87.2	41.1	63.8
Female	45.3	43.3	47.6	40.4	78.3	45.9	23.4	12.8	58.9	36.2
Ethnicity*										
Chinese	75.9	82.5	75.2	68.4	79.8	74.0	86.5	69.1	74.0	86.6
Malay	12.2	6.0	8.9	17.5	10.1	14.7	7.6	21.9	17.7	3.4
Indian	8.7	8.3	10.6	10.1	7.0	8.5	5.4	7.9	7.2	7.5
Others	3.3	3.2	5.4	4.0	3.2	2.8	0.5	1.2	1.1	2.6
Marital status*										
Never married	30.2	28.2	34.5	32.3	37.4	34.3	20.5	15.4	15.1	18.5
Married	62.5	75.0	61.9	61.1	51.1	55.8	75.0	75.0	67.3	74.4
Divorced/separated	5.6	7.5	2.6	5.6	9.3	7.0	3.2	7.5	7.6	7.1
Widowed	1.8	2.2	1.0	1.0	2.1	2.8	1.4	2.2	10.0	0.0

	All occupation (4021)	Legislators, senior officials and managers (658)	Professionals (696)	Associate professionals and technicians (649)	Clerical support workers (365)	Service and sales workers (909)	Craftsmen and Related Trades Workers (89)	Plant and machine operators and assemblers (392)	Cleaners, labourers and related workers (214)	Business owners (49)
Education*										
Primary and below	11.9	0.8	0.9	6.8	2.0	20.1	32.7	30.0	63.1	7.0
Secondary	19.9	13.1	6.7	19.3	18.2	28.7	35.1	37.9	25.1	20.4
Pre-U/Junior College	4.8	2.8	4.2	2.8	7.6	7.3	5.2	4.7	3.1	7.2
Vocational Institute/ITE	7.3	3.5	5.3	13.1	4.2	8.7	8.8	11.6	5.0	10.2
Diploma	21.0	26.5	19.9	24.9	31.2	20.0	10.0	8.0	0.7	18.1
University	35.1	53.2	63.0	33.2	36.9	15.2	8.2	7.8	2.9	37.1
Household Income*										
Below 2000	10.4	1.3	2.3	7.9	6.2	18.1	13.8	26.0	41.7	5.7
2000–3999	19.7	7.1	11.1	21.7	25.1	28.0	32.2	31.6	26.8	17.7
4000–5999	22.7	18.2	22.9	28.2	21.8	23.4	22.0	27.2	16.1	21.7
6000–9999	24.1	28.2	32.1	26.0	25.2	18.5	21.1	11.9	13.4	25.1
10,000 and above	23.1	45.3	31.5	16.3	21.7	12.0	11.0	3.3	2.0	29.7

*Significant differences in proportions at *p* < 0.001 between occupational groups, χ^2^ test

### Prevalence of mental and physical disorders across occupational groups

The lifetime prevalence of mental disorders among participants across occupational groups is shown in Table 3. The prevalence of nicotine dependence was significantly different (*p* < 0.001) across the occupational groups. Mood and anxiety disorders were observed to be the most prevalent mental disorders among ‘Clerical support workers’ while alcohol use disorder and nicotine dependence were more prevalent among ‘Business owners’ and ‘Plant and machine operators and assemblers’, respectively. However, after controlling for socio-demographic variables, ‘Clerical support workers’ were more likely to have anxiety disorder compared to ‘Associate professionals and technicians’ (OR = 2.31, CI 1.02, 5.23, *p* = 0.04) (refer to Table 4).

**Table 3:** Prevalence of lifetime mental disorders across occupational categories

Lifetime mental disorders	All occupations		Legislators, Senior officials and managers		Professionals		Associate professionals and technicians		Clerical support workers		Service and sales workers		Craftsmen and related trades workers	
	%	*n*	%	*n*	%	*n*	%	*n*	%	*n*	%	*n*	%	*n*
Mood disorder	8.4	345	8.4	57	10.2	70	8.4	58	11.4	38	7.6	80	3.9	5
Anxiety disorder	5.2	209	5.2	26	5.6	32	3.8	31	7.8	24	6.1	65	0.3	1
Alcohol use disorder	5.3	234	4.4	34	4.8	30	6.4	43	3.9	19	5.5	51	3.8	6
Nicotine dependence*	4.0	210	2.4	20	1.7	9	4.2	39	1.4	9	5.2	55	3.1	4

Lifetime mental disorders	Plant and machine operators and assemblers		Cleaners, labourers and related workers		Business owners	
	%	*n*	%	*n*	%	*n*
Mood disorder	5.8	21	5.3	13	7.3	3
Anxiety disorder	4.8	21	3.1	8	0.8	1
Alcohol use disorder	8.2	31	4.4	14	14.8	6
Nicotine dependence*	11.7	52	7.2	21	4.2	1

*Significant differences at *p* < 0.001 between occupational groups, χ^2^ test

**Table 4:** Odds of having lifetime mental disorders across occupational categories

Occupational categories	Mood disorder				Anxiety disorder				Alcohol use disorder				Nicotine dependence			
	OR	95% Confidence interval		*p* value	OR	95% Confidence interval		*p* value	OR	95% Confidence interval		*p* value	OR	95% Confidence interval		*p* value
Associate professionals and technicians	Ref				Ref				Ref				Ref			
Legislators, senior officials and managers	0.96	0.53	1.75	0.898	1.57	0.74	3.32	0.235	0.71	0.34	1.48	0.361	1.06	0.44	2.56	0.893
Professionals	1.16	0.65	2.05	0.619	1.53	0.73	3.17	0.258	0.82	0.40	1.70	0.596	0.97	0.35	2.69	0.950
Clerical support workers	1.21	0.64	2.30	0.557	2.31	1.02	5.23	0.044*	1.05	0.43	2.58	0.907	0.86	0.29	2.55	0.790
Service and sales workers	0.86	0.49	1.52	0.603	1.97	0.97	4.02	0.061	0.80	0.41	1.54	0.498	0.83	0.41	1.70	0.614
Craftsmen and related trades workers	0.60	0.14	2.55	0.490	0.14	0.02	1.13	0.065	0.33	0.08	1.42	0.137	0.36	0.08	1.74	0.206
Plant and machine operators and assemblers	0.98	0.44	2.19	0.959	2.44	0.93	6.43	0.071	0.62	0.28	1.38	0.244	1.44	0.71	2.94	0.316
Cleaners, labourers and related workers	0.70	0.22	2.20	0.543	0.86	0.27	2.74	0.803	0.75	0.26	2.15	0.590	1.19	0.44	3.26	0.730
Business owners	0.43	0.06	3.08	0.397	0.36	0.04	2.85	0.330	2.39	0.41	13.85	0.333	2.41	0.19	31.18	0.501

OR was derived using logistic regression analyses after controlling for age, gender, ethnicity, marital status, education and income

*OR* odds ratio, *p<0.05

Table 5 describes the lifetime prevalence of chronic conditions among the different occupational groups. Hypertension (*p* < 0.001), diabetes (*p* < 0.001) and chronic pain (*p* = 0.02) have significant differences across the occupational groups. Hypertension and hyperlipidemia were notably more prevalent among ‘Cleaners, labourers and related workers’. The highest prevalence of diabetes and chronic pain was seen among ‘Plant and machine operators and assemblers’, and ‘Business owners’ respectively. After controlling for socio-demographic variables, ‘Legislators, senior officials and managers’ (OR = 2.06, CI 1.06, 4.01, *p* = 0.03) were more likely to have diabetes compared to ‘Associate professionals and technicians’ (refer to Table 6).

**Table 5:** Prevalence of lifetime chronic conditions across occupational categories

Lifetime chronic conditions	All occupations		Legislators, senior officials and managers		Professionals		Associate professionals and technicians		Clerical support workers		Service and sales workers		Craftsmen and related trades workers	
	%	*n*	%	*n*	%	*n*	%	*n*	%	*n*	%	*n*	%	*n*
Hypertension**	18.7	800	19.2	120	15.4	110	17.0	131	12.2	51	18.7	190	18.4	19
Hyperlipidaema	19.4	799	16.1	115	17.4	113	18.3	124	21.2	65	19.9	188	24.6	23
Diabetes**	7.2	438	6.9	53	3.5	45	4.9	58	4.5	33	7.4	115	12.6	14
Asthma	11.9	514	8.4	63	14.5	101	13.1	88	15.3	56	13.1	127	11.1	9
Chronic pain*	18.9	770	22.9	147	19.2	153	18.4	115	20.8	81	16.7	147	3.4	8

Lifetime chronic conditions	Plant and machine operators and assemblers		Cleaners, labourers and related workers		Business owners	
	%	*n*	%	*n*	%	*n*
Hypertension**	29.8	104	31.5	66	17.8	9
Hyperlipidaema	25.3	100	25.4	60	22.4	11
Diabetes**	16.0	70	14.9	43	13.2	7
Asthma	7.6	36	9.4	29	12.2	5
Chronic pain*	16.1	63	18.3	41	28.9	15

^*^Significant differences at *p* < 0.05

^**^Significant differences at *p* <0.001 between occupational groups, chi-square test

**Table 6:** Odds of having lifetime chronic conditions across occupational categories

Occupational categories	Hypertension				Hyperlipidemia				Diabetes			
	OR	95% Confidence Interval		*p* value	OR	95% Confidence Interval		*p* value	OR	95% Confidence Interval		*p* value
Associate professionals and technicians	Ref				Ref				Ref			
Legislators, senior officials and managers	1.27	0.79	2.05	0.330	0.73	0.46	1.17	0.193	2.06	1.06	4.01	0.033*
Professionals	1.27	0.77	2.10	0.346	1.22	0.76	1.97	0.403	1.05	0.50	2.22	0.897
Clerical support workers	0.76	0.42	1.39	0.380	1.62	0.97	2.72	0.067	1.17	0.55	2.49	0.683
Service and sales workers	0.98	0.62	1.54	0.915	1.03	0.67	1.56	0.901	1.15	0.62	2.14	0.660
Craftsmen and related trades workers	0.67	0.27	1.65	0.382	1.08	0.48	2.41	0.849	1.82	0.63	5.23	0.267
Plant and machine operators and assemblers	1.12	0.68	1.84	0.659	0.94	0.56	1.58	0.818	1.52	0.78	2.97	0.221
Cleaners, labourers and related workers	1.20	0.61	2.37	0.593	0.71	0.36	1.40	0.321	1.09	0.47	2.52	0.846
Business owners	0.88	0.27	2.89	0.829	0.95	0.33	2.78	0.931	3.40	1.03	11.26	0.045*

OR was derived using logistic regression analyses after controlling for age, gender, ethnicity, marital status, education and income

*OR* odds ratio, *p<0.05

### Health-related quality of life and work productivity

Table 7 shows the mean scores of the health-related quality of life domains (physical component score and mental component score) across occupational groups. Higher means were observed in the mental component scores when compared to the physical component scores across the occupational groups. We conducted a linear regression and observed that ‘Craftsmen and related trades workers’ (β = 1.79, 95% CI 0.52; 3.07, *p* < 0.01) were significantly associated with higher health-related quality of life in mental component score (MCS) compared to ‘Associate professionals and technicians’.

**Table 7:** Health-related quality of life across occupational categories

Occupational category	Mean ± SE	Physical component score (PCS)					Mental component score (MCS)			
		β	95% Confidence interval		*p *value	Mean ± SE	β	95% Confidence interval		*p *value
Associate professionals and technicians	54.63 ± 0.25	Ref				55.02 ± 0.33	Ref			
Legislators, senior officials and managers	54.56 ± 0.24	− 0.12	− 0.74	0.50	0.709	56.06 ± 0.28	0.73	-0.20	1.67	0.125
Professionals	54.91 ± 0.24	− 0.22	− 0.86	0.43	0.513	55.41 ± 0.30	0.07	-0.61	1.26	0.495
Clerical support workers	54.03 ± 0.38	− 0.17	− 0.89	0.54	0.635	55.36 ± 0.41	0.50	-0.62	1.62	0.380
Service and sales workers	53.45 ± 0.25	0.01	− 0.61	0.63	0.965	55.54 ± 0.28	0.23	− 0.73	1.19	0.640
Craftsmen and related trades workers	54.70 ± 0.50	− 0.58	− 1.67	0.50	0.293	57.03 ± 0.47	1.79	0.52	3.07	0.006*
Plant and machine operators and assemblers	53.48 ± 0.39	0.46	− 0.35	1.26	0.268	55.84 ± 0.39	0.02	− 1.18	1.21	0.976
Cleaners, labourers and related workers	52.84 ± 0.51	− 0.43	− 1.38	0.52	0.378	56.43 ± 0.60	0.91	− 0.73	2.54	0.277
Business owners	53.88 ± 0.67	1.16	− 0.93	3.25	0.277	54.61 ± 1.14	− 1.22	− 3.84	1.54	0.401

β was derived using linear regression analyses after controlling for age, gender, ethnicity, marital status, education and income, *p <0.01

The differences by occupation in the work-loss days and work-cutback days did not reach statistical significance (Table 8), nevertheless, variations were seen across the occupational groups. The highest work-loss (mean 1.11) and work-cutback (mean 0.75) days due to health was reported among ‘Business owners’ and ‘Professionals’ respectively.

**Table 8:** Work days lost and cut down across occupations due to mental and physical health conditions

Occupational category	Work-loss days in the past 30 days									Work-cutback days inthe past 30 days								
	Quartiles									Quartiles								
	Mean	SD	Min	Max	5th	25th	50th	75th	95th	Mean	SD	Min	Max	25th	5th	50th	75th	95th
Legislators, senior officials and managers	0.25	1.42	0.00	20.00	0.00	0.00	0.00	0.00	1.00	0.38	1.59	0.00	20.00	0.00	0.00	0.00	0.00	2.00
Professionals	0.22↓*	1.09	0.00	15.00	0.00	0.00	0.00	0.00	2.00	0.75↑**	3.48	0.00	30.00	0.00	0.00	0.00	0.00	3.00
Associate professionals and technicians	0.44	2.18	0.00	30.00	0.00	0.00	0.00	0.00	2.00	0.59	2.97	0.00	30.00	0.00	0.00	0.00	0.00	3.00
Clerical support workers	0.51	2.91	0.00	30.00	0.00	0.00	0.00	0.00	3.00	0.56	2.21	0.00	30.00	0.00	0.00	0.00	0.00	3.00
Service and sale workers	0.57	3.37	0.00	30.00	0.00	0.00	0.00	0.00	2.00	0.48	2.57	0.00	30.00	0.00	0.00	0.00	0.00	3.00
Craftsmen and related trades workers	0.81	4.15	0.00	30.00	0.00	0.00	0.00	0.00	1.00	0.09	0.80	0.00	14.00	0.00	0.00	0.00	0.00	2.00
Plant and machine operators and assemblers	0.25	1.81	0.00	30.00	0.00	0.00	0.00	0.00	2.00	0.20	1.63	0.00	30.00	0.00	0.00	0.00	0.00	2.00
Cleaners, labourers and related workers	0.35	1.65	0.00	14.00	0.00	0.00	0.00	0.00	2.00	0.49	2.83	0.00	30.00	0.00	0.00	0.00	0.00	5.00
Business owners	1.11↑**	4.56	0.00	30.00	0.00	0.00	0.00	0.00	1.00	0.08↓*	0.38	0.00	4.00	0.00	0.00	0.00	0.00	0.00

*Lowest value, **Highest value

## Discussion

One of the main objectives of this paper was to examine the associations between occupational groups and socio-demographic characteristics. The differences in age, gender, ethnicity, marital status, educational level and household income across occupational groups are consistent with reports from studies conducted in other populations (Lim et al. 2000; Kessler et al. 2006; De Graaf et al. 2012).

### Prevalence of mental disorders across occupational groups

The overall lifetime prevalence of mood disorders among the employed was 8.4% and it was the most frequently endorsed category of disorders in this sample. Sanderson and Andrews (2006) conducted a structured review of epidemiological studies in workplaces and similarly found depression to be the most prevalent disorder among the employed. Mood and anxiety disorders tend to be more commonly reported (Kawakami et al. 1996; Andrea et al. 2004; Linden and Muschalla 2007; Cohidon et al. 2009) in workplace-related surveys and notably office job workers report elevated levels of anxiety and depression (Kang et al. 2016). A similar observation was made in the current study sample, whereby office job workers (i.e., “Legislators, senior officials and managers”, “Professionals”, “Associate professionals and technicians” and “Clerical support workers”) reported higher rates of mood and anxiety disorders. This could be attributed to long working hours and the job scope as office workers tend to have highly demanding jobs with limited support and resources which become potential risk factors for mental health problems (Karasek 1979; Kang et al. 2016). Andrea et al. (2004) found that subclinical anxiety was associated with jobs with increased psychological demands and conflicts with supervisors while subclinical depression was associated with low decision latitude and decreased social support. Tak (2002) reported a similar finding stating that workplace conflict such as, conflict between departments, and decision-making were the most critical factors contributing to job stress among office workers. Our study further substantiates these findings as ‘Craftsmen and related trades workers’ (i.e., manual workers) reported significantly higher MCS score compared to ‘Associate professionals and technicians’ (office job workers).

Nicotine dependence was observed to be the highest among ‘Plant and machine operators and assemblers’ which coincides with the findings of Siahpush et al., (2006) in a panel study comprising 2000 adult smokers from each of four countries: the United States, Canada, United Kingdom, and Australia. Higher rates of nicotine dependence were largely found among blue-collar professions and those with lower levels of education (Lee et al. 2004; Pennanen et al. 2014). Alcohol use disorder, on the other hand, has been associated with “upper managers” (Marchand 2008; Parker 1992) which has been similarly noted in our study sample among ‘Business owners’. Zhang and Snizek (2003) discussed how job independence and satisfaction have negligible effect on alcohol use and on the contrary, steady employment has the most distinct harmful effect on alcohol use. Different work positions may contribute to the various rates of alcohol use; upper managers tend to view it as a way to unwind after a day’s work or engage in alcohol consumption for work-related networks (Järvinen et al. 2014). Upper managers tend to have a higher income level and this is associated with increased drinking patterns which may be partly explained by the fairly high prices of alcoholic beverages in Singapore (Lim et al. 2007).

### Prevalence of physical disorders across occupational groups

Whilst mental disorders were predominantly associated with skilled office workers, hypertension and diabetes was found to have the highest prevalence among those who engage in heavy manual work; ‘Cleaners, labourers and related workers’ and ‘Plant and machine operators and assemblers’, respectively (Nakamura 2000; Smith 1997). Previous studies that have mainly looked at metabolic syndrome between manual and non-manual workers have consistently reported that manual workers tend to be at a higher risk of the condition due to lower education and poorer socioeconomic status that is linked to poor health practices such as unhealthy dietary habits, lack of exercise, unhealthy behaviours such as smoking and poor health literacy (Sanchez-Chaparro et al. 2008; Gupta et al. 2012). On the contrary, chronic pain was highly prevalent among business owners which has been similarly observed among Japanese white-collar workers with Wakaizumi et al. (2017) reporting an increasing trend in the prevalence of chronic pain. Although pain has been traditionally associated with manual workers (Bernard et al. 2011) recent studies have posited that stressors (commonly found among white-collared workers) such as low job control with high job demands and job stress that is not adequately reciprocated with rewards (i.e. promotion, salary raise, praise) are predictors of chronic pain (Herr et al. 2015).

### Work productivity

With all these in play, the effect of the mental and physical conditions on work-loss days was notably the highest among ‘Business owners’ and lowest among ‘Professionals’. As for work-cutback days, we found that it was highest among ‘Professionals’ and lowest among ‘Business owners’. The plausible reason for these observations could be that that those in the ‘Professionals’ category might be more able than others to slacken at work in response to their health problems and refrain from being absent at work more often. This could be because those in the ‘Professionals’ category could be more adept at withholding their work-cutback days from their supervisors or the nature of their work might make it easier for them to do so and also possibly they are less prone to have supervisors compared to other workers. Business owners on the other hand might have subordinates to handle majority of the workload and might feel more at ease to be away from work for a longer duration due to health problems.

The cross-sectional design of this study brings forth a key limitation of the study; the incapability to assess causality. In addition, we were unable to determine the temporal association of the work-loss and work-cutback days and occupation with health conditions as we assessed them at different time-points. Occupation was assessed at the point of study entry, and lifetime prevalence was analysed for the health conditions whilst work-loss and work-cutback days were based during the 30 days prior to the interview. Collection of the afore-mentioned data at the point of study entry would help us to further narrow down the conditions that affect work productivity and in turn help to curate better policies to tackle this issue. The study sample may also not be representative of Singapore’s population in terms of statistics which shows that ‘Associate Professionals and Technicians’ are generally the biggest occupation group in Singapore in 2019 (SingStat 2020) while in the current study 'service and sales worker' were the largest group. All information in this study was obtained by self-report and this introduces potential the study.

## Conclusion

This is one of the few representative studies which have provided a current comprehensive overview of mental and physical orders among the employed in Singapore. The study has shown that there are differences in the socio-demographic characteristics and prevalence of mental and physical disorders across occupational categories. These health disparities among occupations provide policymakers and researchers with vital information to pilot effective interventions that can improve the psychosocial and physical conditions at work. While occupations with higher odds of mental and physical disorders may be characterized by increased levels of job demands and lack of job security, decreased odds of these disorders among occupations are probably due to high levels of job discretion, good employee-employer relationship and clearly defined job scope (Stansfeld et al. 2011). Policies should be designed to lessen the impact of physical and mental disorders on employees which would contribute to a reduction in work-loss and work-cutback days. The findings also highlight the importance of investigating the causal relationship between the health conditions and work conditions, and identifying and reducing the risk factors experienced by employees. Targeted surveillance and health promotion programs can then be conducted for at‐risk occupations.

## Data Availability

The data that support the findings of this study are available from the corresponding author upon reasonable request.

## Declarations

### Research involving human and animal participants

All procedures performed in studies involving human participants were in accordance with the ethical standards of the institutional and/or national research committee (National Healthcare Group Domain Specific Review Board 2015/01035) and with the 1964 Helsinki declaration and its later amendments or comparable ethical standards. This article does not contain any studies with animals performed by any of the authors.
